# Proximal factors influencing the likelihood of married and cohabiting women in Sierra Leone to use contraceptives. A cross-sectional study

**DOI:** 10.1186/s40834-024-00269-9

**Published:** 2024-02-27

**Authors:** Augustus Osborne, Camilla Bangura

**Affiliations:** Department of Biological Sciences, School of Basic Sciences, Njala University, PMB, Freetown, Sierra Leone

**Keywords:** Intention, Contraceptives, Reproductive health, Women, Sierra Leone

## Abstract

**Background:**

In the tapestry of reproductive health in Sierra Leone, where maternal mortality remains a poignant thread, understanding contraceptive use intentions among married and cohabiting women emerges as a vital motive. This study examines the intention to use contraceptives among married and cohabiting women in Sierra Leone.

**Methods:**

The study analysed the 2019 Sierra Leone Demographic and Health Survey data. A total of 7846 married and cohabiting women comprised the study. A multivariable binary regression analysis was used to examine the predictors of intention to use contraceptives. The regression results were presented using an adjusted odds ratio (AOR) with 95% confidence intervals (CI).

**Results:**

The proportion of intention to use contraceptives among married and cohabiting women was 47% in Sierra Leone. Married and cohabiting women living in the eastern region (AOR = 1.65, 95% CI = 1.18, 2.30), southern region (AOR = 1.45, 95% CI = 1.04, 2.01), secondary education (AOR = 1.42, 95% CI = 1.00, 2.01), listen to the radio at least once a week (AOR = 1.30, 95% CI = 1.08, 1.55), and four or more births (AOR = 2.97, 95% CI = 2.23, 3.96) had higher odds of being associated with intention to use contraceptives. The propensity to utilise contraceptives among married and cohabiting women in Sierra Leone declined as their age increased, especially women aged 45–49 (AOR = 0.07, 95% CI = 0.04, 0.11) who had the least intention of using contraceptives. Married women (AOR = 0.53, 95% CI = 0.39, 0.72) and women who read magazines or newspapers at least once a week(AOR = 0.61, 95% CI = 0.36, 1.o4) had lower odds of contraceptive use intention.

**Conclusion:**

The findings of this study indicate that there is a moderate yet encouraging intention to use contraception among married and cohabiting women in Sierra Leone. Factors like residing in the eastern and southern regions, having secondary education, having more children and regular radio listening are associated with higher contraceptive use intentions. Older women, especially those nearing the end of their childbearing years, have the lowest intention. Married women and regular magazine or newspaper readers were less likely to intend to use contraceptives. These findings call for targeted interventions focusing on rural areas, women with lower education, and older women.

## Introduction

Utilising contraceptives is a very efficient method to avoid unplanned births, decrease mother and child mortality rates, and enhance the overall health and welfare of women and their families [1, 2].. However, many women in sub-Saharan Africa (SSA) face barriers to accessing and using contraceptives, such as lack of information, availability, affordability, social norms, and partner opposition [1].

Out of the 1.9 billion women aged 15–49 worldwide in 2019, approximately 1.1 billion needed family planning [2]. This means that they either currently use contraceptives, with 842 million using modern methods and 80 million using traditional methods or have an unmet need, with 190 million women having a desire to avoid pregnancy but not use any form of contraception [2]. In 2019, the percentage of women who get their family planning needs fulfilled by modern means, as measured by Sustainable Development Goals indicator 3.7.1, was 76% [2].

Sierra Leone is one of the countries in SSA with a low contraceptive prevalence rate and a high unmet need for family planning [3]. According to the 2019 Sierra Leone Demographic and Health Survey (SLDHS), only 21% of married or cohabiting women aged 15–49 were using any method of contraception, and 28% had an unmet need for family planning [3]. These figures indicate that there is a gap between the demand and supply of contraceptive services in the country and that many women are at risk of unintended pregnancies and their associated complications.

Previous studies [4–14] have found that the determinants of contraceptive practice are multifaceted and encompass various factors such as awareness of contraceptive methods, socio-demographic variables (including age, education, religion, income level, marital status, employment), parity, number of living children, access to reproductive health information, frequency of antenatal visits, history of terminated pregnancies, prior HIV testing, place of residence (rural or urban), literacy, sexual activity, communication and approval from partner.

Previous studies in Sierra Leone [10, 15–18] have looked at contraceptive use and the factors that influence its use, such as socio-demographic characteristics, access to services, and quality of information and counselling. Nevertheless, there has been no research conducted on the factors that influence the likelihood of married and cohabiting women in Sierra Leone to take contraception. This country faces multiple challenges, such as poverty, gender inequality, low literacy, and high maternal mortality [16]. Hence, comprehending the factors that influence the inclination to utilise contraceptives among married and cohabiting women in Sierra Leone can facilitate the development and execution of efficient measures to enhance contraceptive adoption and diminish the prevalence of unfulfilled family planning requirements. Therefore, this paper aims to fill this gap by examining the predictors of intention to use contraceptives among married and cohabiting women in Sierra Leone, using the 2019 Sierra Leone demographic health survey.

## Methods

### Data source and design

The 2019 Sierra Leone Demographic and Health Survey (SLDHS) data was used for this study. SLDHS was conducted over four months (from May 2019 to August 2019) to gather data on demographic, health, and nutritional factors among women, children, and men [3]. A cross-sectional design was adopted for the SLDHS, and respondents were sampled using a multistage sampling method. In the first stage, 578 enumeration areas (EAs), consisting of 214 urban and 364 rural regions, were selected. A systematic selection procedure was employed for the second stage to select 24 households from each EA. This selection process ultimately resulted in a sample size of 13,872 households [3]. A detailed description of the survey methodology is available in the literature [3]. The SLDHS study included 15,574 women aged 15 to 49 in Sierra Leone. In the survey, 9837 women were married and cohabiting. This study included 7846 married and cohabiting women with complete datasets in the final analysis. The dataset was accessed following the procedures outlined on the official DHS program website [19]. The study followed the Strengthening Reporting of Observational Studies in Epidemiology (STROBE) guidelines [20].

### Study variables

#### Outcome variable

The outcome variable was the intention to use contraceptives. This variable quantifies the degree to which individuals who do not already use contraceptives intend to utilise any contemporary method in the future. The variable was obtained from the question, ‘Do you intend to use a method to delay or avoid pregnancy at any time in the future?‘. Response options to this question were ‘use later’, ‘unsure about use’ and ‘does not intend to use’. Women who intended to use contraceptives were considered to “Intend to use”, coded as 1, while those who did not intend to use contraceptives were considered “Do not intend to use”, coded as 0. Previous studies [4–6] that utilised the DHS datasets employed the same coding scheme.

#### Explanatory variables

Thirteen explanatory variables were included in the study. The variables include the age of the women, place of residence, region, educational attainment of women and their partners, wealth index, marital status, employment status of women and their partners, parity, and exposure to media (newspapers/magazines, radio, and television). These variables were selected based on their statistically significant association with the use of contraceptives from previous studies [4–6] and their availability in the SLDHS. Table 1 shows the categories of the variables included in the study.

### Data analysis

The data was analysed using SPSS version 28. Percentages were used to present the proportion of married and cohabiting women who intended to use contraceptives and their distribution across the explanatory variables. A chi-square test of independence was conducted to determine the variables significantly associated with the intention to use contraceptives at *p* < 0.05. The variance inflation factor (VIF) was used to test for evidence of collinearity among the variables studied. The results showed that the highest and lowest VIFs were 2.47 and 1.05. Hence, there was no evidence of high collinearity among the variables. Later, binary logistic regression analysis was performed to examine the variables associated with the intention to use contraceptives. Two models were used. The first model was the bivariable analysis that examined the independent association between each explanatory variable and the intention to use contraceptives. The last model was the multivariable analysis containing all the explanatory variables. The results were presented using adjusted odds ratio (AOR) with their respective 95% confidence interval (CI). Statistical significance was set at *p* < 0.05.

## Results

### Proportion of intention to use contraceptives among married and cohabiting women in Sierra Leone

**Fig. 1:**
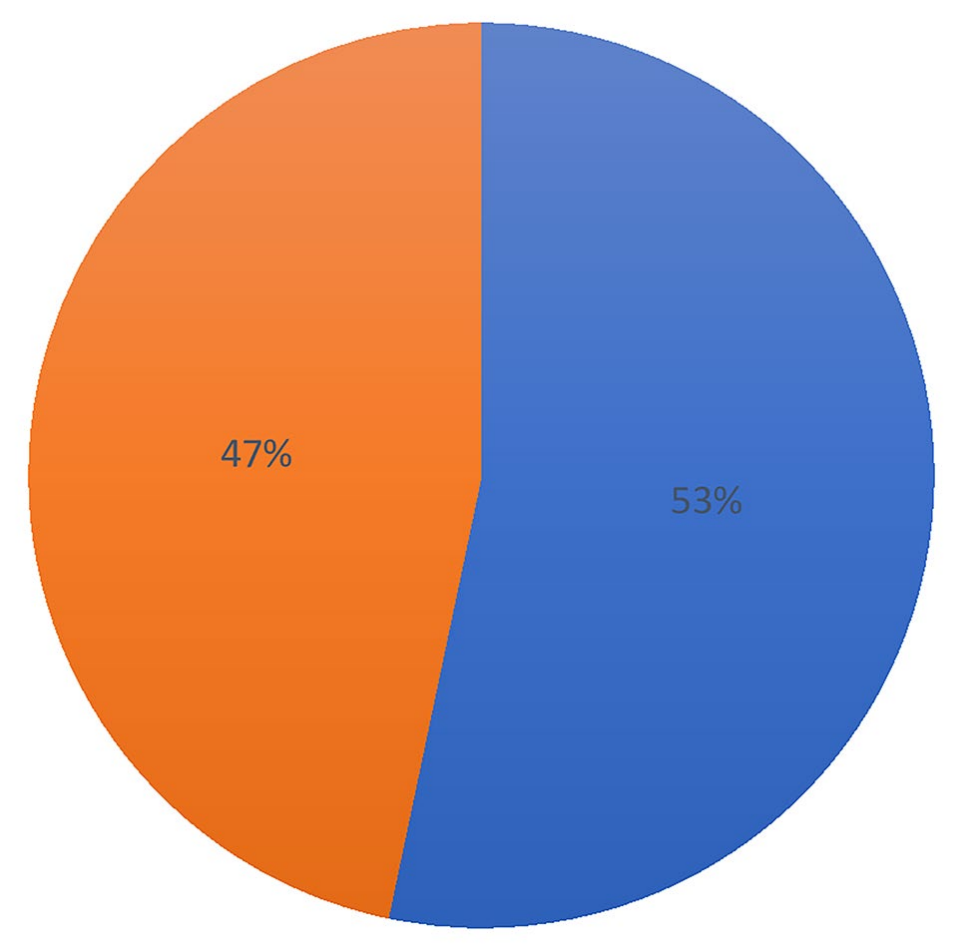
Proportion of intention to use contraceptives among married and cohabiting women in Sierra Leone

Figure 1 shows the results of the proportion of married and cohabiting women who intended to use contraceptives. The results indicated that 47% of Sierra Leonean married and cohabiting women intended to use contraceptives.

### Distribution of intention to use contraceptives among married and cohabiting women in Sierra Leone

Table 1 shows the intention to use contraceptive distribution across the background characteristics of the women in Sierra Leone. In Sierra Leone, married and cohabiting women aged 25–29 had the highest intention to use contraceptives (26.8%), while those aged 45–49 (3.5%) had the least intention to use contraceptives. Married and cohabiting women living in rural areas had the highest intention to use contraceptives (62.7%), while those living in urban areas(37.3%) had the least intention to use contraceptives. Married and cohabiting women with no education had the highest intention to use contraceptives (55.2%), while those with higher education(3.2%) had the least intention to use contraceptives. Married women had the highest intention to use contraceptives (91.6%), while those cohabiting (8.4%) had the least intention to use contraceptives. Married and cohabiting women who were employed had the highest intention to use contraceptives (78.7%), while those unemployed (21.3%) had the least intention to use contraceptives. Married and cohabiting women with four or more births had the highest intention to use contraceptives (39.2%), while those with no birth (6.1%) had the least intention to use contraceptives. Except for region, all the remaining explanatory variables were significantly associated with the intention to use contraceptives at *p* < 0.05.

**Table 1:** Distribution of intention to use contraceptives among married and cohabiting women in Sierra Leone (*n* = 7846)

Variables	Category	Intention to use contraceptives		*P*-value
		No n(%)	Yes n(%)	
Age				< 0.001
	15–19	189(4.4)	237(6.4)	
	20–24	457(10.9)	668(18.7)	
	25–29	611(14.0)	916(26.9)	
	30–34	621(13.9)	674(18.8)	
	35–39	867(19.8)	667(18.8)	
	40–44	712(16.6)	243 (6.9)	
	45–49	867(20.3)	117(3.5)	
Place of residence				0.015
	Urban	1137(32.3)	1198(37.3)	
	Rural	3187(67.7)	2324(62.7)	
Region				0.079
	Eastern	723(18.6)	734(21.6)	
	Northwestern	842(20.8)	607(17.7)	
	Northern	1320(24.8)	858(21.5)	
	Southern	940(18.3)	841(19.6)	
	Western	499(17.5)	482(19.6)	
Women’s Education Level				< 0.001
	No education	3269(72.8)	2034(55.2)	
	Primary	432(10.5)	496(15.3)	
	Secondary	513(13.5)	886(26.2)	
	Higher	110(3.1)	106(3.2)	
Wealth index				0.016
	Poorest	1123(23.6)	843(21.8)	
	Poorer	1091(23.5)	791(21.9)	
	Middle	976(21.3)	712(19.5)	
	Richer	644(16.2)	646(18.1)	
	Richest	490(15.4)	530(18.7)	
Marital status				< 0.001
	Married	4192(96.6)	3261(91.6)	
	Cohabiting	132(3.4)	261(8.4)	
Women’s occupational status				< 0.001
	Unemployed	661(16.3)	732(21.3)	
	Employed	3663(83.7)	2790(78.7)	
Frequency of reading newspaper or magazine				< 0.001
	Not at all	4195(96.3)	3343(93.8)	
	Less than once a week	66(1.9)	134(4.8)	
	At least once a week	63(1.9)	45(1.4)	
Frequency of listening to radio				< 0.001
	Not at all	2889(64.1)	2059(55.8)	
	Less than once a week	616(15.0)	673(20.4)	
	At least once a week	819(20.9)	790(23.8)	
Frequency of watching television				< 0.001
	Not at all	3663(81.5)	2757(75.7)	
	Less than once a week	344(8.9)	409(13.0)	
	At least once a week	317(9.6)	356(11.3)	
Parity				< 0.001
	No birth	264(6.5)	209(6.1)	
	One birth	581(13.4)	579(16.6)	
	Two births	649(15.2)	658(19.4)	
	Three births	682(15.6)	670(18.7)	
	Four or more births	2148(49.2)	1406(39.2)	
Husband/partner’s education level				< 0.001
	No education	3061(67.4)	1969(53.4)	
	Primary	268(6.5)	274(8.0)	
	Secondary	721(18.7)	981(29.7)	
	Higher	274(7.4)	298(8.9)	
Husband/partner’s occupational status				0.025
	Unemployed	300(7.6)	194(6.0)	
	Employed	4024(92.4)	3328(94.0)	

### Predictors associated with the intention to use contraceptives among married and cohabiting women in Sierra Leone

Table 2 shows the determinants associated with the desire for more children among women in Sierra Leone. Married and cohabiting women living in the eastern region (AOR = 1.65, 95% CI = 1.18, 2.30), southern region (AOR = 1.45, 95% CI = 1.04, 2.01), secondary education (AOR = 1.42, 95% CI = 1.00, 2.01), listen to the radio at least once a week (AOR = 1.30, 95% CI = 1.08, 1.55), four or more births (AOR = 2.97, 95% CI = 2.23, 3.96), three births(AOR = 2.18, 95% CI = 1.67, 2.85), two births (AOR = 1.78, 95% CI = 1.37, 2.32), and one birth(AOR = 1.46, 95% CI = 1.13, 1.88) had higher odds of being associated with intention to use contraceptives. With regards to age, the intention to use contraceptives among married and cohabiting women in Sierra Leone decreased with increasing age, especially women aged 45–49 (AOR = 0.07, 95% CI = 0.04, 0.11) had the least intention of using contraceptives. Married women (AOR = 0.53, 95% CI = 0.39, 0.72) had lower odds of contraceptive use intention than cohabiting women. Married and cohabiting women who read magazines or newspapers at least once a week(AOR = 0.61, 95% CI = 0.36, 1.o4) had lower odds of contraceptive use intention than those who don’t read.

**Table 2:** Predictors associated with the intention to use contraceptives among married and cohabiting women in Sierra Leone

Variables	Category	Intention to use contraceptives	
		COR 95% CI	AOR 95% CI
Age			
	15–19	**Ref.**	**Ref.**
	20–24	1.19*** [0.94, 1.51]	0.94*** [0.73, 1.22]
	25–29	1.33*** [1.04, 1.70]	0.93*** [0.69, 1.25]
	30–34	0.94*** [0.74, 1.19]	0.63*** [0.46, 0.86]
	35–39	0.66*** [0.52, 0.84]	0.44*** [0.32, 0.60]
	40–44	0.28*** [0.21, 0.38]	0.17*** [0.12, 0.25]
	45–49	0.12*** [0.08, 0.17]	0.07*** [0.04, 0.11]
Place of residence			
	Urban	**Ref.**	**Ref.**
	Rural	0.80** [0.66, 0.95]	0.89 [0.63, 1.26]
Region			
	Eastern	1.03[0.81, 1.31]	1.65*** [1.18, 2.30]
	Northwestern	0.76 [0.57, 1.01]	1.10 [0.76, 1.61]
	Northern	0.77 [0.57, 1.05]	1.18 [0.79, 1.76]
	Southern	0.95 [0.76, 1.20]	1.45** [1.04, 2.01]
	Western	**Ref.**	**Ref.**
Women’s Education Level			
	No education	0.73^*^[0.53, 1.00]	0.95[0.64, 1.40]
	Primary	1.40* [0.99, 1.99]	1.23 [0.80, 1.90]
	Secondary	1.87*** [1.39, 2.53]	1.42** [1.00, 2.01]
	Higher	**Ref.**	**Ref.**
Wealth index			
	Poorest	0.75^**^ [0.60, 0.95]	0.96 [0.64, 1.44]
	Poorer	0.76** [0.61, 0.95]	0.92 [0.63, 1.34]
	Middle	0.74** [0.60, 0.93]	0.91 [0.64, 1.28]
	Richer	0.91** [0.75, 1.12]	0.90 [0.70, 1.16]
	Richest	**Ref.**	**Ref.**
Marital status			
	Married	0.38*** [0.28, 0.52]	0.53*** [0.39, 0.72]
	Cohabiting	**Ref.**	**Ref.**
Women’s occupational status			
	Unemployed	**Ref.**	**Ref.**
	Employed	0.72*** [0.60, 0.85]	0.95 [0.78, 1.15]
Frequency of reading newspaper or magazine			
	Not at all	**Ref.**	**Ref.**
	Less than once a week	2.65 [1.90, 3.68]	1.63[1.10, 2.43]
	At least once a week	0.75*** [0.47, 1.19]	0.61***[0.36, 1.04]
Frequency of listening to radio			
	Not at all	**Ref.**	**Ref.**
	Less than once a week	1.56*** [1.33, 1.83]	1.30** [1.08, 1.55]
	At least once a week	1.30 [1.12, 1.51]	1.27 [1.04, 1.56]
Frequency of watching television			
	Not at all	**Ref.**	**Ref.**
	Less than once a week	1.55** [1.26, 1.92]	1.18[0.92, 1.51]
	At least once a week	1.27 [1.03, 1.56]	1.05 [0.80, 1.39]
Parity			
	No birth	**Ref.**	**Ref.**
	One birth	1.32 [1.02, 1.70]	1.46*** [1.13, 1.88]
	Two births	1.36*** [1.07, 1.73]	1.78*** [1.37, 2.32]
	Three births	1.27*** [1.02, 1.59]	2.18*** [1.67, 2.85]
	Four or more births	0.85*** [0.68, 1.05]	2.97*** [2.23, 3.96]
Husband/partner’s education level			
	No education	**Ref.**	**Ref.**
	Primary	1.55*** [1.24, 1.94]	1.24 [0.98, 1.56]
	Secondary and above	2.00 [1.74, 2.30]	1.34 [1.15, 1.57]
	Higher	1.51** [1.20, 1.90]	1.22 [0.93, 1.59]
Husband/partner’s occupational status			
	Unemployed	**Ref.**	**Ref.**
	Employed	1.28** [1.03, 1.59]	1.17 [0.91, 1.50]

## Discussion

Our study examined the intention to use contraceptives among married and cohabiting women in Sierra Leone. The study found that 47% of married and cohabiting women in Sierra Leone intend to use contraceptives. The findings from our study are higher than the overall prevalence of intention to use contraceptives in sub-Saharan Africa [4] but lower than the previous studies in Ghana [21] and Ethiopia [7]. The possible explanations for the moderate and promising intention to use contraceptives among married and cohabiting women in Sierra Leone may be due to recent efforts to increase family planning awareness and education through both national campaigns and community-based initiatives, which could be leading to a shift in attitudes and willingness to consider contraception [10]. Women may be increasingly interested in spacing out births or limiting family size for various reasons, including economic considerations, improved child health outcomes, or pursuing personal goals [22]. The expanding access to family planning services and the availability of a broader range of contraceptive methods, particularly in rural areas, could make it easier for women to obtain and use contraception [23]. Radio broadcasts, social media campaigns, and other communication channels promoting family planning messages and positive narratives about contraception might be reaching women and influencing their opinions [10].

The findings from this study revealed that married and cohabiting women living in eastern and southern regions are associated with higher contraceptive use intentions. The findings from our study are consistent with the previous study in Sierra Leone [10]. Possible explanations are rural areas like the eastern and southern regions might have faced historical neglect in terms of healthcare infrastructure. However, recent interventions or targeted programs focused on these regions could have improved access to family planning services and information, contributing to higher contraceptive use intentions [23]. Local initiatives and community-based organisations working on family planning awareness and education might be more active in the eastern and southern regions, particularly if these regions were identified as having lower baseline family planning knowledge or utilisation. This could lead to increased knowledge and positive attitudes towards contraception among women, eventually translating into higher intentions to use it.

Married and cohabiting women with secondary education were associated with higher contraceptive use intentions in Sierra Leone. This finding is consistent with previous studies in Ethiopia [14] and Malawi [9]. Women with secondary education typically have more comprehensive information access, including reproductive health and family planning. This exposure can equip women with accurate knowledge about contraceptive methods, their benefits and side effects, dispelling myths and misconceptions that might deter others from using them [24]. Educated women are more likely to be exposed to media campaigns and educational programs promoting family planning, further enhancing their understanding and awareness of contraceptive options [25]. Education often empowers women, enhancing their confidence and decision-making autonomy within their relationships. This can enable them to communicate their desire for family planning and negotiate contraception use with their partners, overcoming potential social pressures or cultural barriers.

The study revealed that married and cohabiting women who listen to the radio at least once a week were associated with higher contraceptive use intentions. This finding is consistent with a previous study in Ethiopia [26] and Sierra Leone [10]. Radio broadcasts, particularly those dedicated to health and development topics, often disseminate information about different contraceptive methods, their benefits and side effects, dispelling myths and misconceptions. Regular listeners to such programs could gain valuable knowledge and become more open to considering contraception as a viable option [27]. Government-sponsored campaigns or NGO initiatives promoting family planning frequently utilise radio as a primary outreach tool. These campaigns provide targeted messages and information to increase awareness and positive attitudes towards contraceptive use, impacting those who regularly tune in [27]. It’s crucial to acknowledge that radio access and program content might vary across different regions and demographics in Sierra Leone. While this study identified a positive association, the impact might differ based on the specific programs women listen to and the broader context of their communities.

Our study found that married and cohabiting women with one to four or more children were associated with higher contraceptive use intention. This finding is consistent with the previous study in SSA [4]. Having experienced pregnancy and childbirth firsthand, women may become more aware of the demands of childrearing and the importance of child spacing for their health and well-being. This can lead to a greater desire to use contraceptives to space births and ensure healthier intervals between pregnancies [22]. Financial pressures and resource allocation become major concerns with one or more children already present. Women may view contraception as a way to manage family size and ensure adequate resources for the existing children’s education, healthcare, and overall well-being [22]. After becoming mothers, women might experience a shift in their priorities and aspirations. They may wish to pursue further education, career goals, or personal ambitions that frequent pregnancies could hinder. Contraception can then be viewed as empowering them to achieve these goals and pursue their desired future.

With regards to age, the intention to use contraceptives among married and cohabiting women in Sierra Leone decreased with increasing age, especially women aged 45–49 who had the least intention of using contraceptives. This finding is consistent with the previous studies in Malawi, Nigeria, and SSA [4, 5, 9]. As women approach menopause, their natural fertility declines significantly. This might lead to a reduced need for contraception due to the lower probability of conception [5, 9]. Some women in this age group might experience health complications or side effects from certain contraceptive methods, making them less willing to use them [5]. Some older women might hold outdated beliefs about contraceptive safety or effectiveness, discouraging them from using them [9].

Our study found that married women had less intention of using contraceptives than their cohabiting counterparts in Sierra Leone. This finding correlates with the previous study in Sierra Leone [10] and SSA [4]. Married women who express a desire for contraception might face stigma or disapproval from family, community members, or their husbands, discouraging them from using it [28]. The present study also revealed that women who read magazines or newspapers at least once a week had lower odds of contraceptive use intention. Newspapers and magazines, particularly local publications, might feature content reflecting dominant traditional beliefs or cultural norms that emphasise large families and prioritise childbearing. This exposure could reinforce existing societal expectations and potentially discourage women from considering contraception.

### Policy and practice implications

The findings from our study offer valuable insights for enhancing contraceptive use among married and cohabiting women in Sierra Leone. Government and partner organisations should focus education and outreach programs on rural areas, women with lower education, and older women to address specific needs and existing information gaps. They should utilise radio broadcasts and potentially text messages to reach wider audiences with family planning information and dispel myths about contraception. They should partner with local communities and traditional leaders to gain their support and address cultural sensitivities to encourage contraceptive use within their communities. They should assess and revise existing policies to ensure they support and do not hinder access to contraceptives and reproductive health information. By implementing these interventions, promoting gender equality, and investing in family planning programs, Sierra Leone can make significant progress towards achieving its reproductive health goals.

### Strengths and limitations

One of the key strengths of the study is the use of the SLDHS data, which is nationally representative, meaning it includes information from a large and diverse sample of married and cohabiting women across Sierra Leone. This allows us to generalise our findings to the broader population confidently. The SLDHS collects data on a wide range of variables related to contraception and reproductive health, including demographics, socioeconomic status, and contraceptive use. This allows us to explore complex relationships between multiple factors and the intention to use contraceptives. This study, however, has some limitations. The SLDHS is a cross-sectional survey which limits our ability to draw causal inferences about the relationships between variables. The SLDHS relies on self-reported data, susceptible to recall bias and misreporting. This can affect the accuracy of your findings, particularly for sensitive topics like contraception.

## Conclusions

This study reveals a moderate but promising contraceptive use intention among married and cohabiting women in Sierra Leone, which indicates progress in family planning awareness and highlights the potential for further increases. Factors like residing in the eastern and southern regions, having secondary education, and regular radio listening are associated with higher contraceptive use intentions. Women with more children show stronger intention to use contraceptives, likely due to a desire for child spacing. Conversely, older women, especially those nearing the end of their childbearing years, have the lowest intention, indicating a need for age-specific messaging and interventions. Married women were less likely to intend to use contraceptives. Additionally, regular magazine or newspaper readers were less likely to intend to use contraceptives. These findings call for targeted interventions focusing on rural areas, women with lower education, and older women. Leveraging radio and digital media, partnering with local communities, ensuring service accessibility, and empowering women are crucial steps to increase contraceptive use. Policy revisions, program investment, and ongoing monitoring are essential for achieving reproductive health goals.

## Data Availability

Data is publicly available via the measure dhs website at https://dhsprogram.com/data/available-datasets.cfm.

## Abbreviations

AOR: Adjusted Odds Ratio
CI: Confidence Interval
COR: Crude Odds Ratio
EA: Enumeration areas
SLDHS: Sierra Leone Demographic and Health Survey
SSA: Sub-Saharan Africa
